# SlitNET: A Deep Learning Enabled Spectrometer Slit

**DOI:** 10.1021/acs.analchem.4c06014

**Published:** 2025-04-29

**Authors:** Youxi Zhang, Ciaran Bench, Preveen Surendranathan, Mads S Bergholt

**Affiliations:** † Centre for Craniofacial and Regenerative Biology, King’s College London, London SE1 9RT, U.K.; ‡ National Physical Laboratory, Hampton Road, Teddington, Middlesex, London TW11 0LW, U.K.

## Abstract

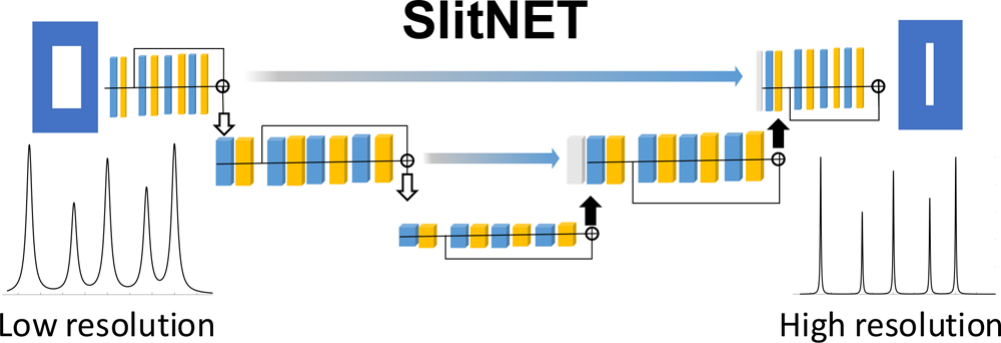

The efficiency and resolution of dispersive spectrometers play crucial roles in optical spectroscopy. Achieving optimal analytical performance in optical spectroscopy requires striking a delicate balance between employing a narrow spectrometer input slit to enhance spectral resolution while sacrificing throughput or utilizing a wider slit to increase throughput at the expense of resolution. Here, we introduce a spectrometer slit empowered by a deep learning model SlitNET. We trained a neural network to reconstruct synthetic Raman spectra with enhanced resolution from low-resolution inputs. Subsequently, we performed transfer learning from synthetic data to experimental Raman data of materials. By fine-tuning the model with experimental data, we recovered high-resolution Raman spectra. This enhancement enabled us to distinguish between materials that were previously indistinguishable when using a wide slit. SlitNET achieved a resolution enhancement equivalent to employing a 10 μm slit size but with a physical input slit of 100 μm. This, in turn, enables us to simultaneously achieve high throughput and resolution, thereby enhancing the analytic sensitivity and specificity in optical spectroscopy. The incorporation of deep learning into spectrometers highlights the convergence of photonic instrumentation and artificial intelligence, offering improved measurement accuracy across various optical spectroscopy applications.

## Introduction

Optical spectroscopy is an indispensable analytical technique utilized across a wide range of scientific disciplines, including materials science, biomedical research, chemistry, and astronomy. By examining the interaction between electromagnetic radiation and matter, it provides detailed insights into the electronic, structural, and dynamic properties of atoms and molecules. In materials science, optical spectroscopy enables precise characterization of material properties, while in biomedical research, it facilitates the noninvasive study of biological tissues and disease diagnostics.

Among the various optical spectroscopy methods, Raman spectroscopy is particularly notable for its ability to provide detailed molecular fingerprints of materials through the measurement of inelastic light scattering.^1^ This unique capability renders Raman spectroscopy an invaluable tool for both fundamental research and practical applications, offering profound insights into the molecular structure of materials. Although Raman spectroscopy offers numerous benefits, it is limited by the inherent weakness of the generated Raman signals, necessitating spectral acquisition times of about 1–10 s per spectrum for many applications. For example, obtaining high spatial resolution Raman spectroscopic imaging in biomedicine often requires several hours, rendering it impractical for high-throughput applications. The light collection efficiency and resolution of dispersive spectrometers are closely interrelated and constitute a key experimental consideration. The resolution of spectrometers is influenced by several factors such as slit width, f-number, diffraction order, grating period/efficacy, and the focal distance of the spherical mirror/lens.^2^ Spectrometer slits are imaged onto the detector and regulate the amount of light entering the instrument, thereby determining both throughput and resolution. An important trade-off arises between these two parameters, as narrower slits enhance the spectral resolution but reduce throughput, whereas wider slits increase throughput at the expense of spectral resolution. This fine balance ultimately determines the analytical sensitivity and specificity of any spectroscopic analysis. This limitation has spurred researchers to explore innovative approaches aimed at surmounting this trade-off and simultaneously enhancing both throughput and resolution.^3,4^

Instrument approaches have been developed utilizing coded aperture with a Hadamard mask for modulating input intensity. This spatial amplitude modulation mask functions as a convolution operator with a two-dimensional orthogonal basis. The sensor captures a coded image, which is subsequently reconstructed using an inverse transform operator. This encoding method leverages the entire large aperture of the mask for optical input, while the spectral resolution of the output is determined by the size of each individual mask element.^5^ The concept of computational high-throughput slits for improving both the effective spectral resolution and efficiency of a Raman spectrometer has also been proposed in the literature.^4^ A numerical deconvolution approach formulated as a probabilistic state problem has been shown to enhance spectral resolution by ∼50% while achieving gains in throughput (>2 times efficiency).^4^ Although this technique has shown improvements in coarse metrics like resolution, such resolution enhancement may not improve the representation of complex Raman spectra used for analytical studies. For Raman spectroscopy, peak shapes largely depend upon interactions between molecules and their environment. A fundamental requirement for a successful approach to model the effect of a slit is that it can separate natural peak broadening caused by sample properties from the instrumental broadening. Enhancing spectra so that this type of peak broadening is separated from instrumental peak broadening is nontrivial for experimental data and cannot straightforwardly be modeled using deconvolution. Analytical modeling in such cases is difficult due to our limited understanding of the underlying physical processes and instrument characteristics. In contrast to peak deconvolution, data-driven approaches based on deep neural networks are versatile and flexible tools that have the capacity to learn highly complex and nonlinear transformations of data.^6−8^ Provided there is sufficient data, deep neural networks have shown enormous potential for resolution enhancement across several domains including microscopy and medical imaging.^9,10^ We recently introduced SpectrAI,^11^ a domain specific python package for spectroscopic analysis (spectral denoising, super-resolution, segmentation, classification, and transfer learning). Spectral denoising and super resolution allowed us to reconstruct Raman spectra/images with high SNR thereby enabling Raman microscopy with 40–140× improvement in speed.^12^ Denoising, image reconstruction, and classification of Raman spectra has been reported by several groups.^12−17^ While much of the previous research has focused on the classification and segmentation of spectral data,^18−20^ the fusion of deep learning and optical instrumentation offers significant potential to enhance both measurement accuracy and efficiency.^21−23^

Here, we introduce a deep learning-enabled spectrometer slit model (SlitNET) that leverages neural networks to reconstruct high-resolution Raman spectra from low-resolution spectra measured using a wide input slit. SlitNET learns the influence of the spectrometer slit, which allows us to improve the spectral resolution while preserving the optical throughput. In turn this enhances the overall spectroscopic performance surpassing the limitations of traditional approaches. To achieve this, first we simulate comprehensive data sets of synthetic Raman spectra and train a robust deep learning model to reconstruct high-resolution spectra from low-resolution inputs. To successfully apply this to real data we perform transfer learning from synthetic data to experimental Raman data. The effectiveness of SlitNET is demonstrated by reconstructing Raman spectra and distinguishing between materials that were indiscernible using a wide spectrometer slit. Hence, we show here that SlitNET offers the simultaneous high-throughput and -resolution opening new avenues across many analytical applications in optical spectroscopy.

## Materials and Methods

### Simulation of Synthetic Low and High-Resolution Raman Spectra

We simulated 100,000 paired Raman spectra with both low and high spectral resolution to create a comprehensive data set for model training and validation. To generate these synthetic Raman spectra, we started by creating a random number of delta functions with random locations across the spectral range. These delta functions served as the initial, idealized spectral peaks. Next, we introduced natural broadening to these peaks by convolving each delta function with Lorentzian kernels. The parameters of these Lorentzian kernels were randomly selected between 3 and 30 cm^–1^ to represent the natural broadening effects caused by intermolecular interactions. A random number of peaks (*n* = 25–50) were chosen across the entire spectrum with random position, full width at half-maximum (fwhm) and intensity. This step ensured that the simulated spectra mimicked the physical broadening observed in real Raman spectra. After applying Lorentzian convolution, we further processed the spectra to emulate the instrumental broadening effects. Specifically, each synthetic spectrum was convolved with a single Gaussian kernel. The Gaussian kernel’s parameters were adjusted to replicate the broadening effects due to the spectrometer slit (see Raman Spectroscopy Instrumentation). For the high-resolution synthetic spectra, which served as the ground truth or target data, we emulated a narrow slit equivalent to a 10 μm slit size (fwhm ∼ 5 cm^–1^). In contrast, for the low-resolution synthetic spectra, which acted as the input data, we used a wider slit equivalent to a 100 μm slit size (fwhm ∼ 19 cm^–1^). These slit sizes were chosen based on our spectrometer configuration to ensure realistic data generation. To further enhance the realism of our synthetic spectra, we added various noise sources. This included signal shot noise, detector dark noise, and CCD readout noise. Noise was assumed to be normal distributed. The standard deviation of shot noise increases with the square root of the signal intensity. The square root of signal intensity at each pixel was calculated and added as the shot noise standard deviation σ_Shotnoise_. For detector noise a random standard deviation of 1% was set to represent both detector noises (including dark current noise σ_Darkcurrent_ and read out noise σ_Readout_). This value was selected to align the synthetic spectra with the experimental spectra with reasonable SNR. The total standard deviation was then calculated as
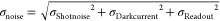


### Raman Spectroscopy Instrumentation

We used a near-infrared (NIR) Raman spectroscopic setup to collect Raman spectra of materials. We employed a 785 nm excitation Raman probe (RPB, InPhotonics Inc., US) using a 500 mW laser excitation source (CleanLaze, B&W Tek Inc., US). The backscattered Raman light was analyzed using an Acton LS785 spectrometer (Teledyne Instruments, US), which was equipped with a thermoelectric cooled (−70 °C) back-illuminated deep depletion Pixis 400 × 1340 charged coupled device (CCD) (Teledyne Instruments). The Acton LS785 featured a variable input slit, which allowed us to adjust the slit width with micrometre precision. This flexibility was essential for controlling the spectral resolution. The Raman scattered light was collected and directed into the spectrometer via a 105 μm diameter optical fiber, which overfilled the entrance slit slightly to ensure maximum light throughput. We focused on acquiring Raman spectra in the fingerprint region between 500 and 1800 cm^–1^. The spectral resolution achieved was ∼19 cm^–1^ with a slit width of 100 μm and ∼5 cm^–1^ with a slit width of 10 μm. These resolutions were determined using an argon–mercury calibration lamp (HG-1, Ocean Insights), which also provided precise reference atomic lines for calibrating the wavelength axis of the spectrometer.

### Raman Spectroscopic Library of Materials

Raman spectroscopy was performed by depositing the materials on a aluminum substrate to reduce background signals. The laser power was (∼493 mW). The integration time (0.5–100 s) was proportionally adjusted for each sample to compensate for the signal reduction when using a narrow slit. A library of 16 arbitrary chosen samples were measured using the wide (100 μm) and narrow (10 μm) entrance slit. The library of 16 samples included urea (Sigma-Aldrich), d-mannitol (Sigma-Aldrich), citric acid (Sigma-Aldrich), glutamic acid (Sigma-Aldrich), d-(+)-glucose (Sigma-Aldrich), glycine (Sigma-Aldrich), l-arginine (Sigma-Aldrich), d-fructose (Sigma-Aldrich), l-methionine (Sigma-Aldrich), fake rubber glass, polypropylene, ethanol, isopropanol, standard translucent resin (Anycubic), stearic acid (Sigma-Aldrich), polyethylenimine (Sigma-Aldrich) and polystyrene. As the purpose of this was merely to create an arbitrary Raman library, we did not control the purities of these materials.

### Data Augmentation for Transfer Learning

We augmented the experimentally obtained data set to enhance the robustness of our analysis. Specifically, a total of 100 pairs of Raman spectra were created computationally. This was achieved by randomly superimposing the Raman spectra of the 16 material samples. For each generated pair, we maintained consistency by assigning the same random percentage of a certain material to both the high- and low-resolution spectra. This ensured that each pair reflected realistic variations in spectral composition while preserving the intrinsic relationships between the different resolution levels. This data augmentation process was pivotal for our study, as it significantly expanded the experimental data set, providing a more diverse range of spectral features.

### SlitNET Model Training, Validation, and Testing

SlitNET was based on ResUNet for spectral resolution enhancement. A synthetic model was trained using data, consisting of pairs of low-resolution spectra and their corresponding high-resolution counterparts. To validate the performance of the model, we employed a stratified data splitting strategy. The training set consisted of 80% of the data, while the validation and independent testing sets each accounted for 10%. The deep learning model was optimized using the Adam optimizer with a learning rate of 0.0001 and a batch size of 128. During transfer learning, batch size was adjusted to 2 and the learning rate was adjusted to 0.0002 to avoid local minima and achieve faster convergence. The model was trained for 100 epochs first and another 100 epochs for transfer learning, with early stopping applied based on the validation loss to prevent overfitting. To evaluate the performance of the model, several metrics were utilized, including the correlation coefficient and the fwhm.

### Data Preprocessing

The experimental Raman spectra underwent preprocessing to ensure data quality and consistency. Initially, each measured Raman spectrum was corrected for its background signal. This correction was tailored to the specific experimental conditions, taking into account the configuration of the slit size, as well as the integration time used during the measurements. Following the background correction, the spectra were subjected to baseline correction by iteratively fitting and subtracting a baseline polynomial function. Lastly, vector normalization was implemented to standardize the spectral intensities across all samples. This normalization process involved scaling the Raman spectra, so they had unit area thus ensuring uniformity across the data set.

### Software Implementation

All experiments and analyses were conducted using the Python programming language. The deep learning model and associated algorithms were implemented using the PyTorch 2.1.0 library, using a NVIDIA TITAN V GPU. For data preprocessing, augmentation, and model training, we utilized our domain-specific Python package, SpectrAI/DeepeR.^24,12^ This package has been tailored specifically for spectroscopy applications, incorporating a suite of tools designed to streamline these processes. SpectrAI facilitates various essential tasks, such as spectral data cleaning, normalization, augmentation, and the preparation of data sets for deep learning models. The implementation of SlitNET is made available at our repository http://github.com/youxizhang1/slitnet and SpectrAI is available at https://github.com/conor-horgan/spectrai.^11^ These repositories include all the necessary code, models and documentation to replicate our experiments and apply the model to new spectral data sets.

## Results

### Training of SlitNET Using Synthetic Raman Data

**Figure 1:**
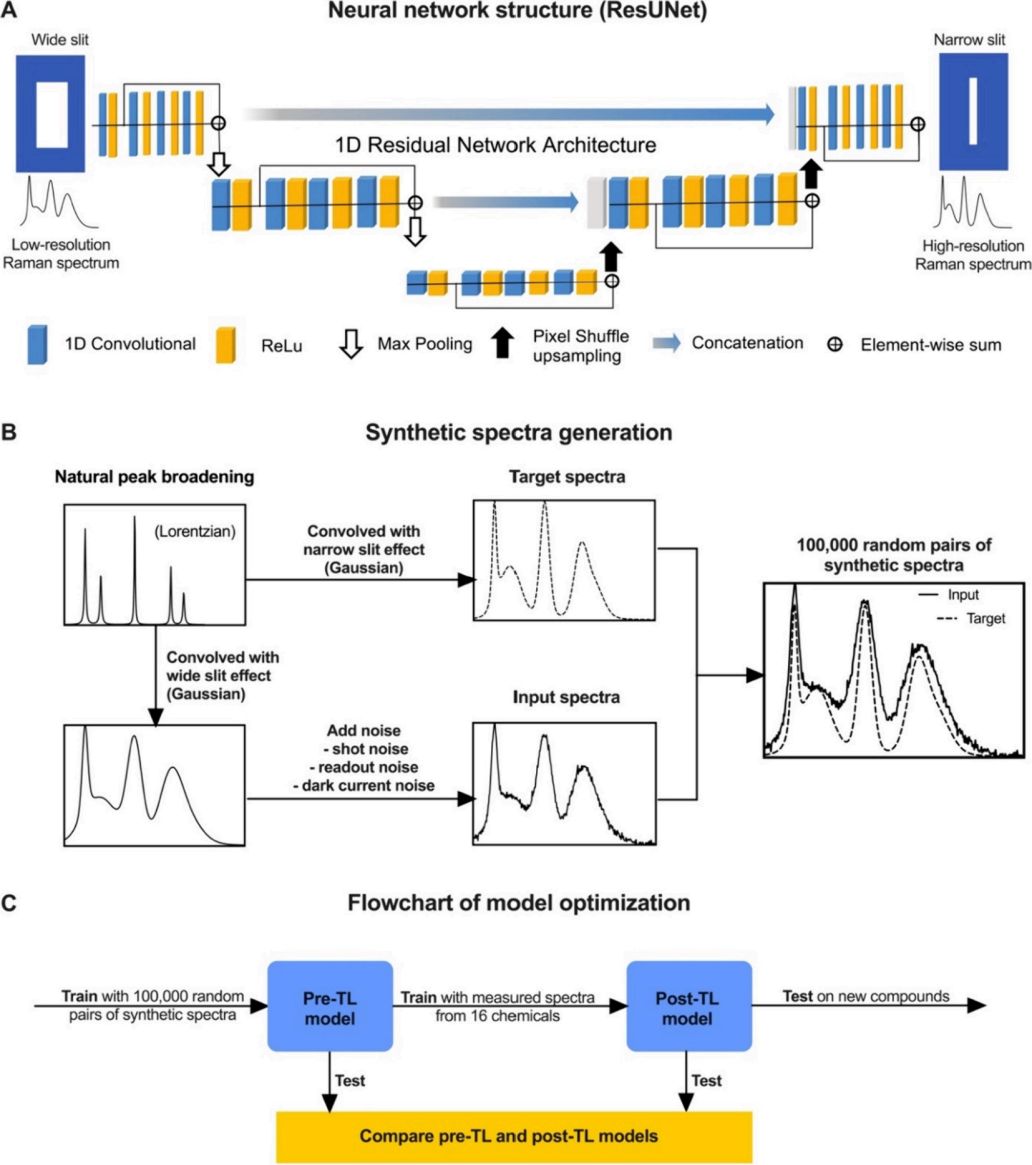
SlitNET deep learning architecture for spectral resolution enhancement. (A) Schematic representation of the ResUNet architecture used in this study. The ResUNet combines residual connections and U-Net-like skip connections to enable effective information flow and feature extraction, facilitating the reconstruction of high-resolution spectra from low-resolution inputs. (B) Random delta function generation and intensities are used for synthetic Raman data generation. These were convolved with Lorenzians to create natural peak broadening. A pair of high-resolution target spectra and low-resolution input spectra were generated by convolving the spectra with different Gaussian functions to simulate a narrow and broad slit. A total 100,000 pairs of synthetic Raman spectra were generated. (C) The workflow developed in this study involves training the model on synthetic Raman data and applying transfer learning to a limited set of experimental spectra to further enhance the model’s performance, accounting for complex spectrometer aberrations.

The deep learning architecture utilized in this study was a ResUNet model adapted from our SpectrAI package^24^ (Figure 1A). This architecture combines residual connections and U-Net-like skip connections, enabling efficient information flow and feature extraction. Given a set of low-resolution spectral inputs *x*_1_, *x*_2_, ···*x*_*i*_ ∈ *X* and their high-resolution counterparts *y*_1_, *y*_2_, ···*y*_*i*_ ∈ *Y*, where the optimal transformation is represented by some nonlinear unknown function that performs the enhancement task *f*(*x*): *X* → *Y*. The ResUNet model *g*_θ_ with parameters θ, learns to approximate this function such that *g*(*x*) ≈ *f*(*x*) by using a training set of paired examples [*x*_*i*_, *y*_*i*_] in a supervised optimization scheme. We introduce a model, SlitNET, which captures slit width across the spectrum as a feature, allowing for the precise reconstruction of high-resolution spectra, even in cases where peaks overlap. We first created comprehensive synthetic data sets of Raman spectra (Figure 1B). In this study, we generated a data set of 100,000 paired high-resolution and low-resolution simulated Raman spectra for the purpose of training a robust model for resolution enhancement. The simulated data sets were designed to emulate our variable spectrometer input slit in our experimental Raman setup (see Materials and Methods). The synthetic data set was designed to encompass a diverse range of realistic spectral characteristics, including varying peak positions, widths and different noise contributions. Briefly, we synthesized low-resolution and high-resolution Raman spectra by randomly convolving a number of delta-functions with different Lorentzian kernels to capture the characteristic shape of natural line shapes. The impact of the varying spectrometer slit width was simulated by convolution the entire spectrum with a Gaussian. This ensures that all Raman peaks are affected by an identical slit broadening effect. The low-resolution spectra were convolved with a Gaussian (fwhm ∼ 19 cm^–1^) corresponding to a 100 μm width slit, and the high-resolution spectra were convolved with a Gaussian (fwhm ∼ 5 cm^–1^) equivalent to a 10 μm width slit. Finally, we introduced noise contributions including shot and detector noise to the data. This comprehensive approach for synthesizing data allowed us to create an arbitrary large data set to train and validate the performance of the deep learning model in reconstructing high-resolution spectra from low-resolution inputs. Here, we found that 100,000 synthetic Raman spectra were sufficient to train a robust model.

**Figure 2:**
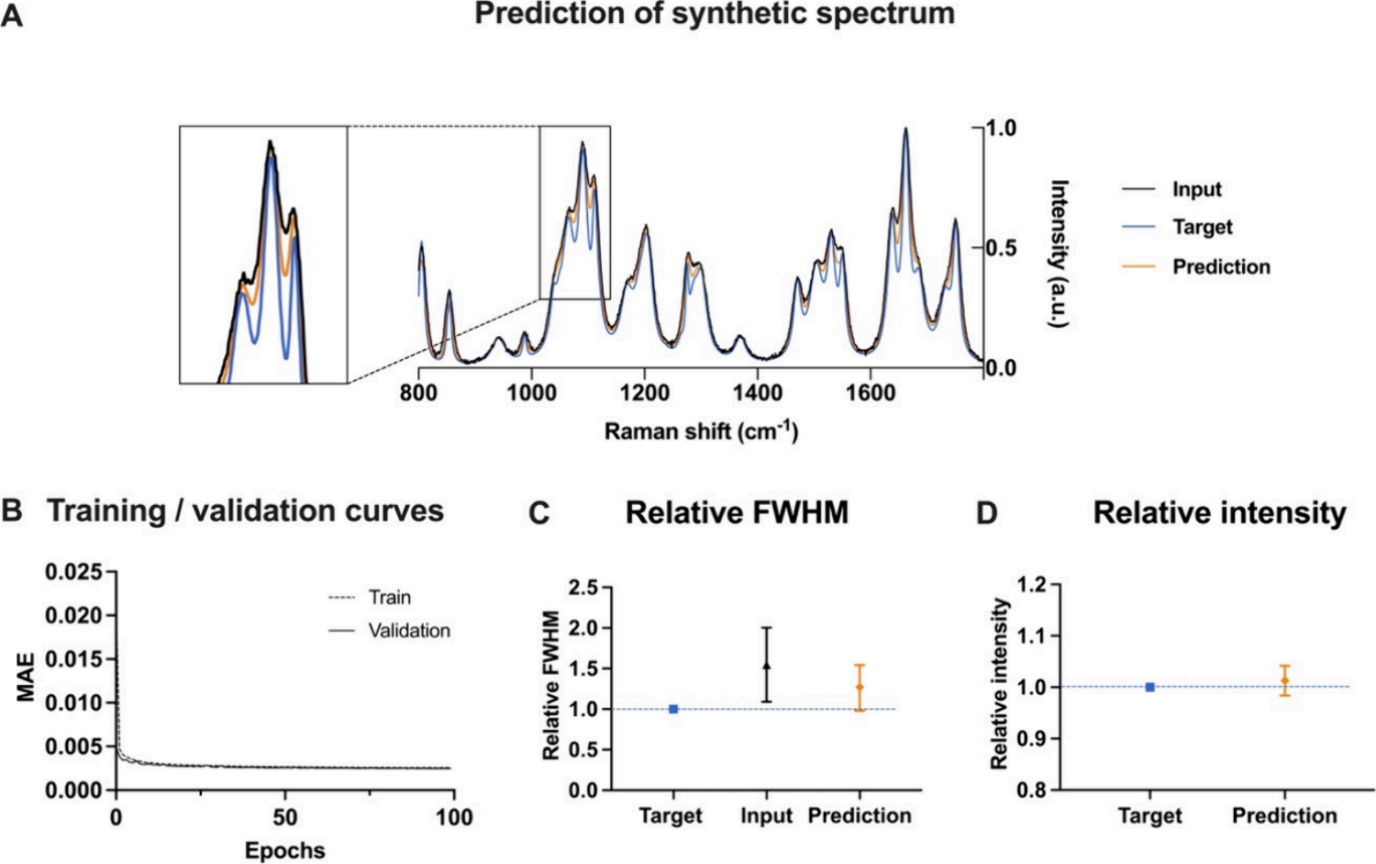
SlitNET application to synthetic Raman data. (A) A comparison of the low-resolution input spectra (black), their corresponding target high-resolution spectra (blue), and the spectra predicted by the deep learning-enabled spectrometer slit (tangerine). The predicted spectrum more closely resembles the target high-resolution spectrum, indicating the model’s ability for resolution enhancement. (B) Training and validation learning curves during model training. The graph shows the mean absolute error (MAE) plotted against the number of training epochs. (C) The full width at half-maximum (fwhm) indicates that the model can improve the resolution of synthetic spectra (*n* = 5 isolated peaks). (D) Comparison of peak intensities showing less than 2% deviation compared to high-resolution target spectra (*n* = 5 isolated peaks).

The ResUNet was trained using a training (80%) and validation (10%) data set to assess the degree of overfitting. Finally, we benchmarked the performance using the independent testing data set (10%). We trained our model for 100 epochs (6.8h on a NVIDIA TITAN V GPU). We show representative examples of the low-resolution input spectra, target high-resolution spectra, and the spectra predicted by SlitNET (Figure 2A). The predicted spectra more closely resembled the target high-resolution spectra, indicating the capability of the model to learn and recover fine details in the complex synthetic Raman spectra. The loss function over the course of training epochs shows that it effectively learned to reconstruct high-resolution spectra from low-resolution inputs (Figure 2B). These comparisons, demonstrated the effectiveness of the approach in reconstructing high-resolution spectra from low-resolution inputs. We benchmarked the performance of the developed model by calculating the average fwhm (Figure 2C) of 5 isolated peaks that did not exhibit overlap. We finally verified that the predictions preserved the relative peak intensities (Figure 2D).

### Transfer Learning with Experimental Raman Data

**Figure 3:**
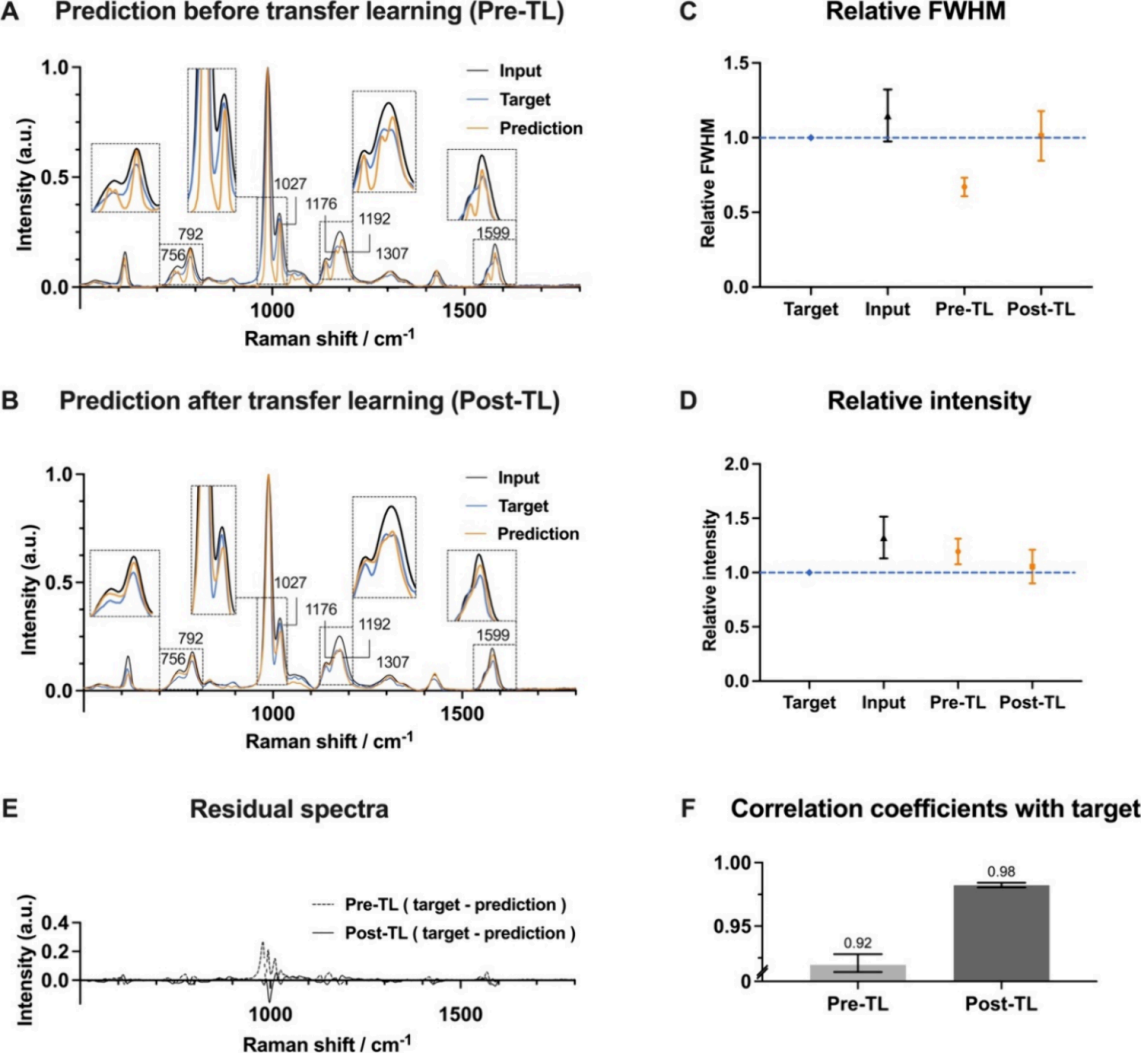
SlitNET transfer learning application to experimental Raman data. (A) Application of synthetic SlitNET model to real Raman spectrum of polystyrene before transfer learning (TL). This illustrates that the model’s capability can enhance the resolution of the spectra but that it overpredicts the resolution for most peaks. (B) TL application significantly improves predictive capability, resulting in spectra closely resembling the high-resolution target spectrum (blue) in terms of both resolution and intensity. (C) Comparison of the full width at half-maximum (fwhm) of Raman peaks relative to the target (*n* = 5 isolated peaks). These data shows that Post-TL the SlitNET model enhances the resolution. (D) Relative intensity in the spectra before and after TL. (E) Residual spectra between Pre- and Post-TL models and the target. The residual spectra show that the Post-TL model more accurately replicate the target. (F) Correlation coefficients of Pre-TL model (0.92) and Post-TL model (0.98) with respect to the target.

We subsequently sought to apply the model, initially developed using synthetic data, on real Raman spectroscopic data (Figure 1C). Experimental Raman spectra are highly complex, reflecting both the molecular structure of the sample and instrumental effects. Raman peaks may exhibit nonsymmetry due to instrumental effects such as smile-curvature aberrations. In certain cases, limited monochromaticity of the laser can contribute to broadening in Raman spectra. Furthermore, experimental Raman spectra have baseline variations and noise contributions, each with distinct statistical properties. The synthetic Raman data belong to the synthetic spectral domain *D*_s_(χ_s_, Υ_s_, *P*(*x*_s_), *P*(*x*_s_, *y*_s_)) composed of an input feature space χ_s_, an output feature space Υ_s_, a marginal distribution *P*(*x*_s_), and a joint distribution *P*(*x*_s_, *y*_s_), where mismatch with the spectral domain of the real test data *D*_y_(χ_t_, Υ_t_, *P*(*x*_t_), *P*(*x*_t_, *y*_t_)) will therefore generally result in poor generalization of *g*_θ_ when applied experimental spectra. Despite these potential sources of spectral domain mismatch, the application of the synthetic model on real Raman data from polystyrene surprisingly showed reasonable predictive capability (Figure 3A). The resolution of Raman peaks was overall enhanced such as (e.g., 792, 1027, 1599 cm^–1^) suggesting that the synthetic data set is representative of experimental Raman data. However, we found that the model tends to overpredict the resolution compared to the narrow-slit Raman spectrum, as well as hallucinating new minor peaks (e.g., near 756 cm^–1^). The predicted peak intensities were also slightly higher than for the target (e.g., near 792 cm^–1^). Overall, the synthetically trained model tends to make fairly accurate predictions, but its performance prior to transfer learning (Pre-TL) remains suboptimal, as expected.

To improve the predictive capability of SlitNET, we applied transfer learning from synthetic to real data. The aim here was to adjust the parameters of *g*_θ_ originally trained to perform the enhancement task τ_s_ on synthetic data described by the domain *D*_s_ so that the adjusted model *g*_θ′_ will perform the analogous task τ_t_ on real spectra from the target spectral domain *D*_t_, where the low resolution spectra acquired with a wide slit *x*_1_′, *x*_2_′, ···*x*_*i*_′ ∈ *X*′ and their corresponding high resolution spectra acquired with a narrow slit *y*_1_′, *y*_2_′, ···*y*_*i*_′ ∈ *Y*′. We trained SlitNET with the same ResNET architecture as *g*_θ_ using the parameters of the pretrained model as the initialization. All other training parameters were kept constant as used for training the synthetic model. We measured high- and low-resolution Raman spectra of 16 arbitrary chosen material and chemical compounds that exhibit well-defined Raman signals (Figure S1). To generate the experimental Raman data set with both high and low resolution, a micrometre-controlled variable slit was used to adjust the slit size to 10 and 100 μm, respectively. We then augmented the data comprehensively (See Materials and Methods) and performed transfer learning on the Raman spectra using our model developed from synthetic data. Finally, we applied the transfer learned model to an independently measured Raman spectrum of polystyrene (Figure 3B). These results demonstrate the ability of the deep learning model to enhance the resolution of real spectroscopic data around peaks such as 756, 1176, and 1599 cm^–1^. We evaluated the performance of the developed model by measuring the fwhm of isolated Raman peaks (Figure 3C) and evaluated the relative intensity preservation (Figure 3D). We also calculated the residual spectra (Figure 3E) and compared the correlation coefficients of the predicted Raman spectra with the target high resolution spectrum (Figure 3F). These results show that by leveraging a transfer-learning the SlitNET model can effectively enhance the resolution of Raman peaks.

**Figure 4:**
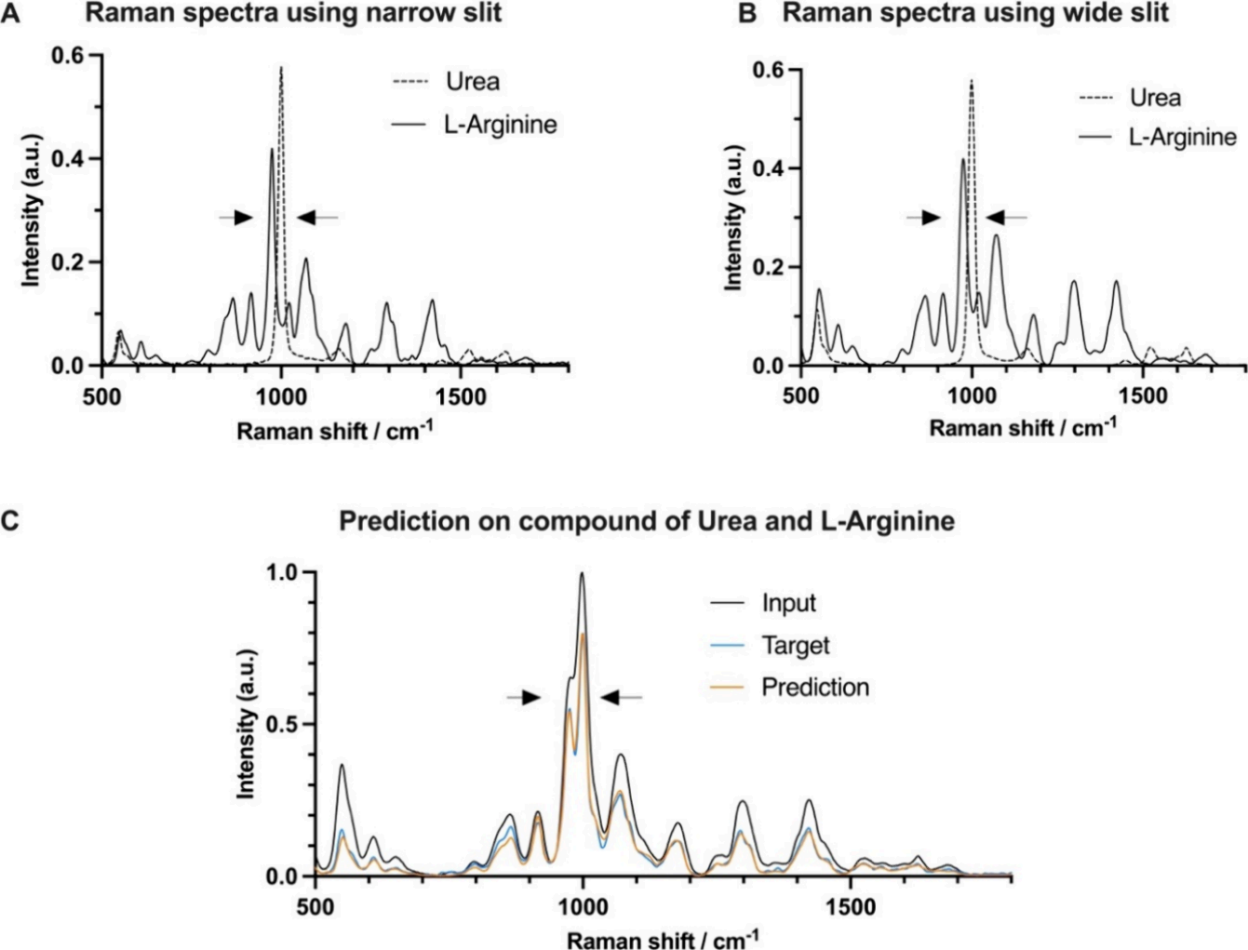
Application of the SlitNET model for identification of two different materials. (A) High resolution Raman spectra of l-Arginine and urea using a narrow slit (10 μm). These spectra show two close peaks located near 970 and 1003 cm^–1^. (B) Low resolution Raman spectra of l-Arginine and urea obtained using a wide slit (100 μm). (C) Wide slit input (100 μm) and target spectra (10 μm) of the superposition of the spectra from the two materials. The predicted spectrum from the input (depicted in tangerine) clearly resolves the overlapping peaks near 970 and 1003 cm^–1^.

While the resolution enhancement is impressive it does not showcase whether the enhancement can effectively aid in analytical tasks such as differentiating overlapping Raman peaks from different molecules. We therefore investigated if the developed SlitNET model can be used to resolve subtle molecular differences that cannot be resolved using a wide slit. We applied the pretrained SlitNET model to Raman spectra of two different molecules (l-arginine and urea) (Figure 4A,B). l-arginine and urea shows close Raman peaks near 970 and 1003 cm^–1^. These Raman peaks are clearly distinguished when measured using a narrow 10 μm slit (Figure 4A) and heavily overlapped when measured using a 100 μm wide spectrometer slit (Figure 4B). We demonstrate here that by applying the transfer learned SlitNET model (Post-TL) to the wide-slit compound spectra that these two peaks can be clearly resolved (Figure 4C). Application of SlitNET to low resolution Raman spectra would therefore allow us to differentiate l-arginine from urea. These results shows that SlitNET enables the discrimination of molecules with close Raman peaks and that the model developed from synthetic data and subsequently retrained on experimental data can improving the resolution and accuracy for new data. To further demonstrate this for a more complex example, we created a mixture spectrum of three different molecules (stearic acid, glycine and l-methionine) with high spectral resolution (Figure S2A) and low spectral resolution (Figure S2B). We then applied the SlitNET model to the Raman spectra showing excellent prediction largely enhancing the Raman spectrum for molecular identification (Figure S2C). While this also could have been achieved using multivariate regression analysis of the library compounds, our results showcase an important analytical scenario where neural networks can directly enhance the performance an optical instrument using a data driven approach. Achieving both high throughput and high resolution simultaneously opens new possibilities for optical spectroscopy, particularly in weak-intensity applications.

## Discussion

We presented SlitNET that can model a dispersive spectrometer slit using a synthetic and experimental data driven approach. This offers major advantages over conventional peak deconvolution methods. While peak deconvolution techniques have been widely used for spectral analysis, they rely heavily on accurate a priori knowledge of the underlying statistics, line shapes and the position/number of peaks present in the spectra. Here we incorporated a physical model through synthetic data set generation and used transfer learning to enable finetuning and accurate application to experimental data. By pretraining the model on large-scale synthetic spectral data sets or leveraging existing pretrained models, the experimental time and computational resources required for model training can be significantly reduced. This facilitates the implementation and deployment of the deep learning-enabled spectrometer slit in practical applications. While the results without transfer learning are promising, our findings suggest that implementing transfer learning is highly recommended, as it substantially enhances spectral reconstruction beyond the simulated model.

SlitNET learns how to convert a low-resolution to a high-resolution spectrum without requiring prior knowledge of the peak positions. This is achieved by spanning a wide range of realistic experimental scenarios during training using synthetic data. This makes the approach robust and less dependent on manual intervention or assumptions about the spectral features. Comparing our approach to existing literature studies, we have achieved improvements in resolution while preserving signal intensity and effectively capturing fine spectral details for Raman data. More importantly, this enables us to enhance the analytical sensitivity (higher throughput) and specificity (higher resolution) of the measurement. We demonstrated that fine-tuning by transfer learning on experimental data further improved the resolvability of peaks by learning of more complex features present in experimental data.

The developed deep learning model even generalized well for complex experimental Raman spectra. Importantly, we did not observe hallucinations from new Raman peaks after transfer learning. It should be noted that the width of the slit is important as there is an inherent loss of information, resulting in a fundamental ambiguity in the problem. When employing a sufficiently wide slit it remains impossible to fully reconstruct high resolution spectra. Consequently, this approach proves effective only if the wide-slit spectra contain adequate information for the network to accurately identify convolution features of the slit. The reconstruction of very low-resolution spectra (e.g., using low density gratings) may not be achievable. Given spectral features at a local level look very similar across spectra acquired from a range of different materials, a large receptive field (i.e., a broad spectrum) is likely needed to ensure as much context as possible is provided to the model, so that it can adequately impose the correct high-resolution representation of features on a given region of the spectrum.

It is also important to note that the generalization capability may vary for unknown or highly complex settings that significantly deviate from the training data. For instance, in biomedical applications, the Raman spectra are highly overlapping and typically consists of exceedingly complex spectra from a myriad of molecules as well as autofluorescence. Further studies are needed to investigate the performance and limitations of our method in such scenarios. There are other limitations of the methodology. Our study focused on Raman spectroscopy in the fingerprint range (i.e., 500–1800 cm^–1^), and the performance of the deep learning-enabled slit may vary for other spectroscopic modalities. The choice of line shapes used in the simulated data set, namely Gaussian and Lorentzian has certain limitations in terms of representation of experimental data. While these line shapes capture common spectral characteristics, they will not fully represent the complexity of optical spectrometers. In some cases, the line shapes may not accurately capture the true underlying physics or instrumental broadening effects (e.g., asymmetric peaks). Different spectrometers might exhibit different characteristics including wavelength range, instrument response and level of stray light. Hence the transfer learning represents an essential component for generalization and high-fidelity spectral reconstruction/enhancement. Flexibility is crucial, enabling adaptation to varying experimental conditions, while robust validation ensures reliable performance. We anticipate that deep learning will have a profound impact on applied spectroscopy in the coming years, not only for resolution enhancement but also for addressing other instrumental challenges, such as standardization, through robust intersystem translation with new intuitive approaches enabled by transfer learning.

## Conclusions

In summary, the deep learning-based computational slit SlitNET enhances the resolution of spectroscopic data. SlitNET effectively addresses challenges in low-light applications. The application of the model to synthetic and real spectroscopy data demonstrated its effectiveness in reconstructing high-resolution spectra and discriminating between materials. The integration of deep learning as a generic part of spectrometer instrumentation signifies a significant advancement in the synergy between artificial intelligence and photonics hardware. Our findings highlight the potential of bundling spectrometers with deep learning models. As we look ahead, future research will focus on enhancing the performance of the network model and testing the applicability and efficiency of our methodology on in various areas from biomedicine, materials science, pharmaceutical research, environmental monitoring and astronomy. We envision that SlitNET can become particularly important for any low light spectroscopic applications such as in biomedical and clinical applications where high spectrometer throughput is often needed.

## Supporting Information Available

Measured spectra of materials through an entrance slit of 10 μm and 100 μm (Figure S1); and application of the SlitNET model for identification of a mixture of three differentmaterials (Figure S2) (PDF)
