# Supplementary material for: SlitNET: A Deep Learning Enabled Spectrometer Slit
Source: Anal Chem. 2025 Apr 29;97(18):9675–82. doi: 10.1021/acs.analchem.4c06014 (PMC12079622; doi:10.1021/acs.analchem.4c06014)
Supplement: Supplementary file 1 — ac4c06014_si_001.pdf [file ac4c06014_si_001.pdf]

Supplementary information SlitNET: a deep learning enabled spectrometer slit

Youxi Zhang[1], Ciaran Bench[2], Preveen Surendranathan[1], Mads S Bergholt[1*]

[1] *Centre for Craniofacial and Regenerative Biology, King's College London, London SE1 9RT, UK*

[2] *National Physical Laboratory, Hampton Road, Teddington, Middlesex, TW11 0LW, UK*

**Corresponding authors*: mads.bergholt@kcl.ac.uk

Table of context

Figure S1 | Measured spectra of materials through an entrance slit of 10 µm and 100 µm

Figure S2 | Application of the SlitNET model for identification of mixture of 3 different materials

Supplementary Figure 1

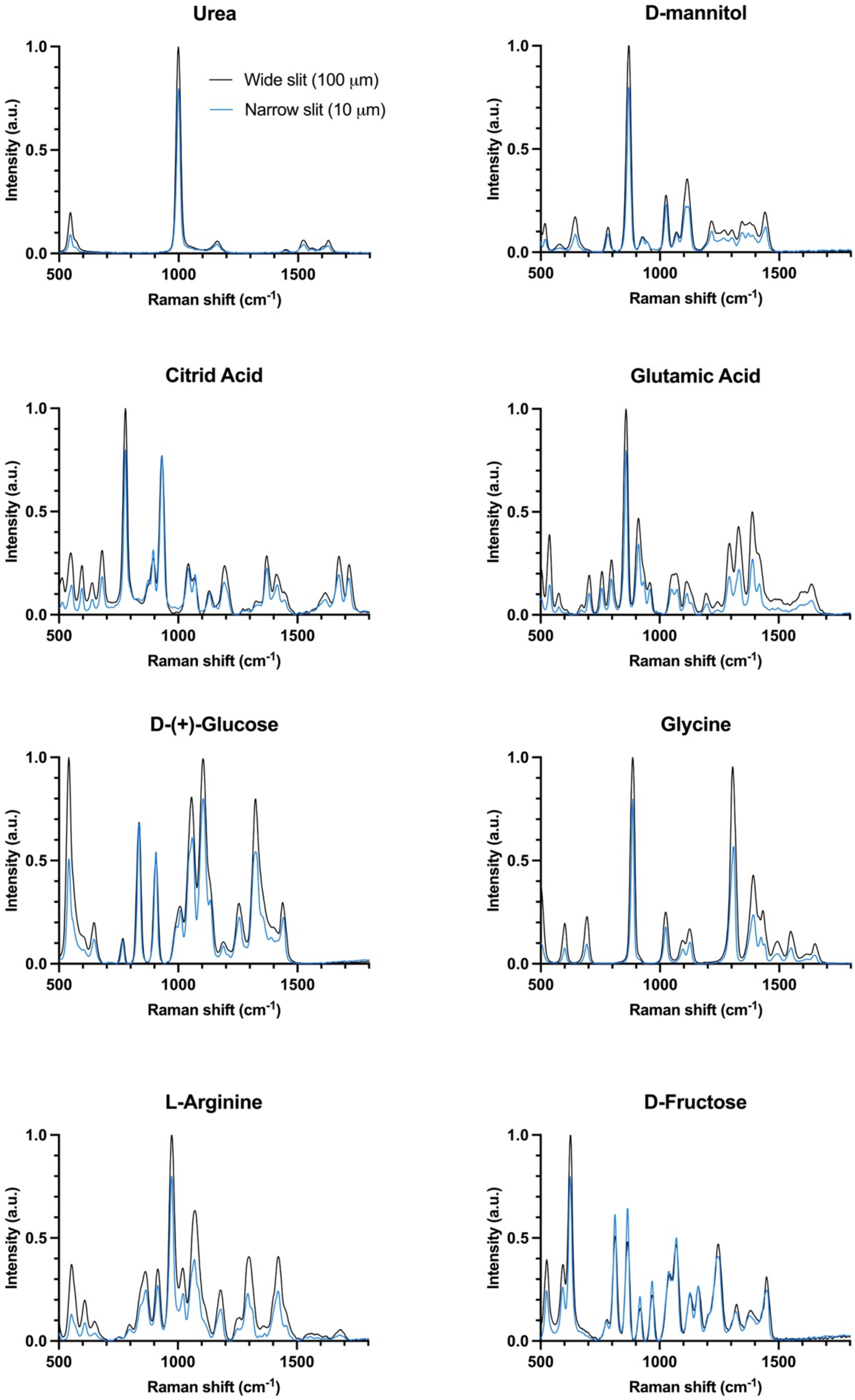

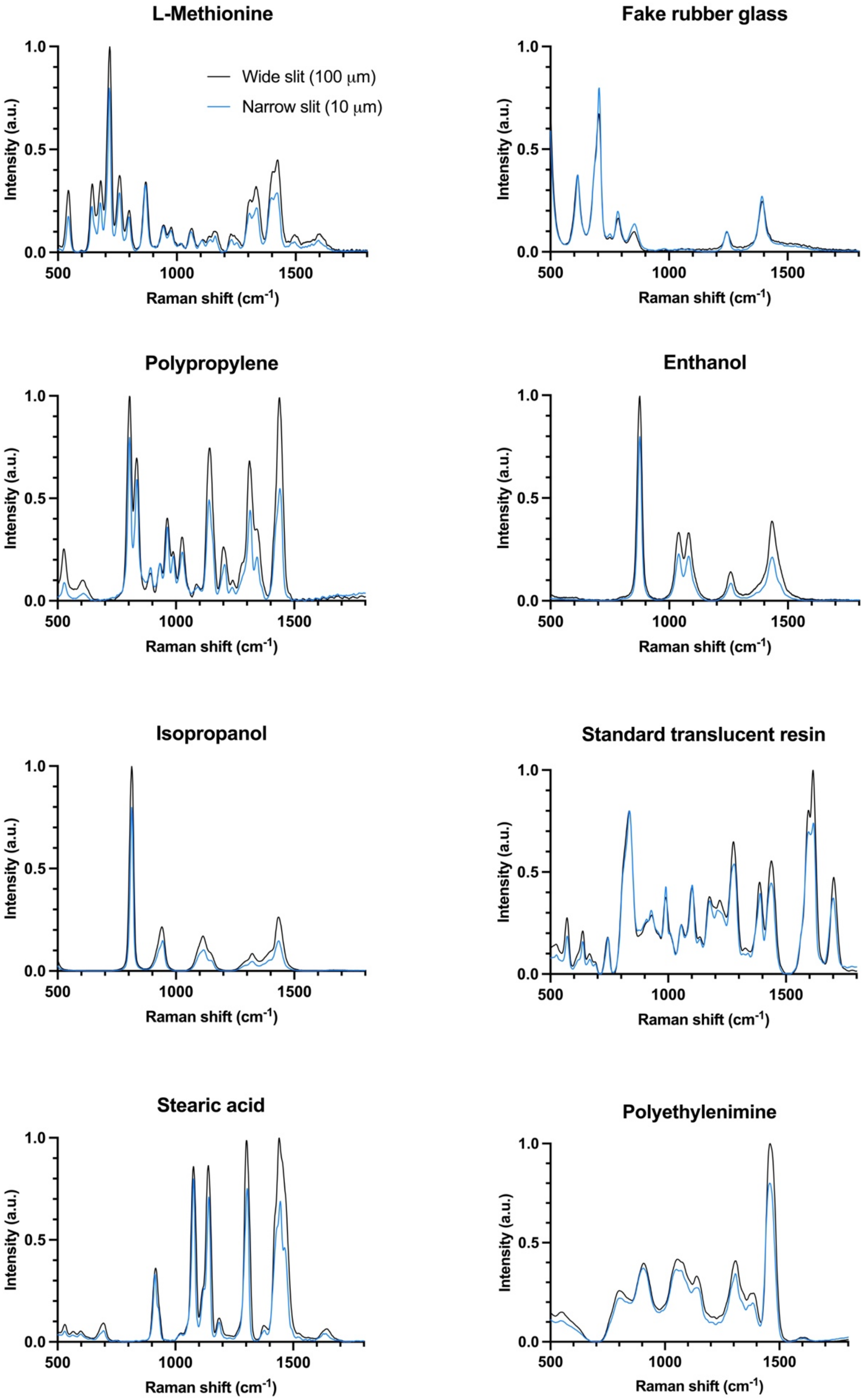

L-Methionine
Wide slit (100 μm)
Narrow slit (10 μm)
Fake rubber glass
Polypropylene
Enthanol
Isopropanol
Standard translucent resin
Stearic acid
Polyethylenimine
Intensity (a.u.)
Raman shift (cm-1)
0.0
0.5
1.0
500
1000
1500

Figure S1 | Measured spectra of materials through an entrance slit of 10 µm and 100 µm. Wide-slit spectra were normalized to 1, and narrow-slit spectra were normalized to 0.8 for visualisation purpose. A total of 16 materials were measured for transfer learning. The library includes D-fructose (Sigma-Aldrich), Sucrose (Sigma-Aldrich), D-(+)-glucose(Sigma-Aldrich), D-serine(Sigma-Aldrich), Glycine (Sigma-Aldrich), Glutamic acid (Sigma-Aldrich) , L-methionine (Sigma-Aldrich), L-isoleucine (Sigma-Aldrich), Citric acid (Sigma-Aldrich), Stearic acid (Sigma-Aldrich), Yeast nitrogen base w/o amino acids (Sigma-Aldrich), D-mannitol (Sigma-Aldrich), Fake rubber glass, Plastics , Polyvinyl chloride, Polypropylene, Agarose base (Sigma-Aldrich), Ethanol, Isopropanol, OCT (CellPath) and Polystyrene (refer to Figure 3).

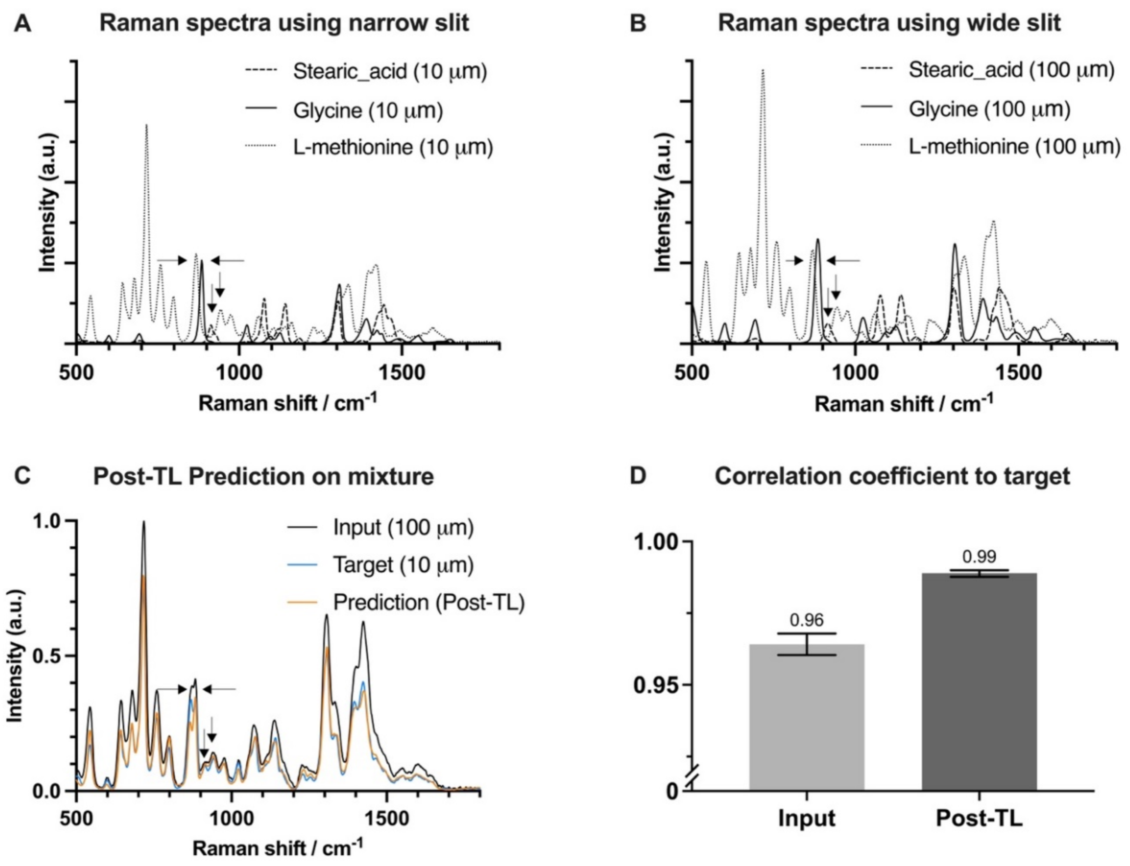

Figure S2 | Application of the SlitNET model for identification of mixture of 3 different materials A) Raman spectra of Stearic acid, glycine and L-methionine shows several close peaks, located at 868 cm^{-1}, 885 cm^{-1}, 916 cm^{-1} and 948 cm^{-1}. All spectra are obtained via narrow slits (10 μm). **B)** Raman spectra of stearic acid, glycine and L-methionine obtained using a wide slit (100 μm). **C)** Wide slit input and target spectra of synthetic compounds obtained by superposition of the spectra from the three materials. The predicted spectrum (depicted in tangerine) clearly resolves two overlapping peaks located around 868 cm^{-1}, ~885 cm^{-1}, ~916 cm^{-1} and ~948 cm^{-1}. **F**) Correlation coefficients of input and predictions , relative to target spectrum are calculated to be 0.96 for input and 0.99 for the Post-TL model.
